# Invited commentary on “Green HEMS in mountain and remote areas: reduction of carbon footprint through drones?”

**DOI:** 10.1186/s13049-023-01120-x

**Published:** 2023-11-06

**Authors:** E. ter Avest, M. Kratz, T. Dill, M. Palmer

**Affiliations:** 1 Air Ambulance Kent, Surrey and Sussex, Redhill, UK; 2 University Medical Center Groningen, University of Groningen, Groningen, Netherlands; 3 Emergency Medical Services, Centre for Prehospital Emergency Care, Department of Emergency, Anaesthesia and Pain Medicine, Tampere University, FinnHEMS 30 & 40, Tampere, Finland; 4 Department of Anaesthesiology and Pain Medicine, University Hospital Bern, Inselspital, Bern, Switzerland; 5 Emergency Medical Transfer Retrieval Service- Wales Air Ambulance, Ty Elusen, Ffordd Angel, Llanelli Gate, Dafen, Llanelli Wales

We’d like to thank the authors for their valuable addition to our article “Green HEMS: How to make it happen”. The authors mention in their article the potential role of drones and unmanned aerial vehicles (UAVs) in mountainous areas. They suggest that drones may substitute helicopters for certain tasks and may contribute to a better and earlier triage of patients who may be in need of a HEMS team.

Although we agree on the potential of drones at large, two things require further consideration. First, although it has been demonstrated that drones are able to deliver vital equipment (such as defibrillators) quickly to scene in remote or inaccessible areas, it remains questionable to which extend this will result in a reduction in HEMS deployment rate, as almost all patients who are in need of this equipment will also need urgent subsequent treatment and expedited transport. Second, the merit of drones to assist in triage decisions has yet to be established and will likely be highly dependent on the prevalence of acute pathology / critically ill patients as well as geography. Especially when travel distances are longer, making the choice to send a drone initially may potentially result in a significant delay in treatment. This risk has to be carefully balanced against the mentioned potential merits.

Finally, the authors mention that in mountainous areas HEMS helicopters are sometimes used for the transport of non-critically ill patients. Although the decision to do so may be impacted by many factors, including availability of ground resources, we agree with the authors that HEMS dispatchers should remain vigilant when it comes to deployment for this purpose: It may not only affect availability of this scarce resource for critically ill patients, but, as the authors rightly point out, also has a significant environmentally impact.

## Data Availability

Not applicable.

